# Absorption of the Tissues of the Mouth from Pressure

**Published:** 1889-04

**Authors:** 


### Absorption of the Tissues of the Mouth from Pressure.

The absorption which occasionally takes place from constant pressure upon the tissues of the mouth was well exemplified by two cases brought forward at the Odontological Society of London recently by Mr. Hern and Mr. E. Lloyd Williams respectively. In the first case the patient had been wearing a set of artificial teeth, the upper and lower of which were attached by means of springs. She had not removed these teeth from her mouth for several years, and as a result, the lower had become buried in the floor of the mouth ; it was also so twisted round as nearly to sever the anterior pillar of the fauces. There was found to be complete anæsthesia of the right half of the tongue and corresponding portion of the floor of the mouth, also complete loss of the sense of taste on that side. There was no pressure on the inferior dental nerve, as sensibility was present in the skin over the chin. The palate was removed with considerable difficulty, and caused much hemorrhage. Some weeks later Mr. Hern observed a whitish cord passing across the gap in the anterior pillar of the fauces; this proved to be the gustatory nerve, and on pinching it with tweezers a tingling sensation was felt in the tip of the tongue. Nine months later sensation had partly returned in the tongue.

Mr. Lloyd Williams showed a man who had extracted two loose molars for himself, the sockets of which probably communicated with the antrum. The patient, being annoyed by the cavity, plugged it with gutta-percha, gradually increasing the size until a plug was used measuring more than an inch in diameter. The hole, however, was much larger than this, as the patient was in the habit of encircling the gutta-percha with a piece of lettuce leaf. The walls of the antrum presented in places foul ulcerated spots, which, however, speedily disappeared when an obturator was placed in the mouth and antiseptic injections were used.—*Lancet*, Jan. 19, 1889.

